# Cardiac (tele)rehabilitation in routine clinical practice for patients with coronary artery disease: protocol of the REHAB + trial

**DOI:** 10.3389/fcvm.2024.1387148

**Published:** 2024-07-29

**Authors:** Rutger F. R. van Mierlo, Vitalis J. G. Houben, Sem A. O. F. Rikken, Juan Jose Gómez-Doblas, Jordi Lozano-Torres, Arnoud W. J. van ’t Hof

**Affiliations:** ^1^ Department of Cardiology, Maastricht University Medical Center (MUMC+), Maastricht, Netherlands; ^2^ Department of Cardiology, Zuyderland Medical Center, Heerlen and Geleen/Sittard, Netherlands; ^3^ Department of Radiation Oncology (Maastro), Research Institute for Oncology and Reproduction (GROW), Maastricht University, Maastricht, Netherlands; ^4^ Department of Cardiology, St. Antonius Hospital, Nieuwegein, Netherlands; ^5^ Department of Cardiology, Cardiovascular Research Institute Maastricht (CARIM), Maastricht University, Maastricht, Netherlands; ^6^ Department of Cardiology, Hospital Universitario Virgen de la Victoria, Málaga, Spain; ^7^ Centro de Investigación en Red de Enfermedades Cardiovasculares (CIBERCV), IBIMA-Plataforma BIONAND, Universidad de Málaga, Málaga, Spain; ^8^ Department of Cardiology, Hospital Universitari Vall D’Hebron, Barcelona, Spain

**Keywords:** cardiac rehabilitation, cardiac telerehabilitation, rehabilitation, quality of life, physical activity, tele health, eHealth, tele monitoring

## Abstract

**Introduction:**

Cardiac rehabilitation programs face the challenge of suboptimal participation, despite being a level Ia recommendation. Cardiac telerehabilitation, with its potential to engage patients who might otherwise not show interest, necessitates the adaption of existing center-based cardiac rehabilitation programs to facilitate rehabilitation at home. REHAB + is a mobile cardiac telerehabilitation program cocreated with patients and rehabilitation centers, aiming to future-proof cardiac rehabilitation and improve accessibility. The REHAB + application enables users to remotely communicate with their coach, receive on-demand feedback on health goal progression, and reduces the need for frequent in-person meetings at the cardiac rehabilitation center. The REHAB + study seeks to compare patient-related outcomes and characteristics of patients between those offered the option to participate in cardiac telerehabilitation and those attending center-based cardiac rehabilitation over a twelve-month period.

**Methods:**

The REHAB + study is a multicenter, prospective, matched controlled, observational study that includes (N)STEMI patients eligible for cardiac rehabilitation. We aim to enroll 300 participants for cardiac telerehabilitation and 600 for center-based cardiac rehabilitation. Participants opting for cardiac telerehabilitation (REHAB+) will be matched with center-based cardiac rehabilitation participants. Additionally, characteristics of patients unwilling to participate in either center-based rehabilitation or telerehabilitation but are willing to share their demographics will be collected. The primary endpoint is quality of life measured with the SF-36 questionnaire at three and twelve months, with patient-related characteristics driving intervention choice as the most important secondary endpoint. Secondary endpoints include physical activity, modifiable risk factors, and digital health experience. The trial is registered at clinicaltrials.gov with registration number NCT05207072.

**Discussion:**

The REHAB + trial is unique by offering patients freedom to choose between cardiac telerehabilitation and center-based rehabilitation. The integration of digital components into cardiac rehabilitation has the potential to complement behavioral change strategies for specific patient groups. Offering patients the option of cardiac telerehabilitation next to center-based rehabilitation could enhance overall cardiac rehabilitation participation rates.

## 1 Introduction

Cardiac rehabilitation (CR) programs reduce the risk of cardiovascular hospitalization, future myocardial infarction, and cardiovascular mortality, while also improving quality of life (1–3). However, participation in CR programs remains low, despite strong evidence that outline their benefit (3–7).

Previous research (8–11) outlined various categories that might discourage patients from participation, which include practical, systemic, personal and COVID-19 related barriers. Practically, patients might lack the means to commute to the CR center, lack the time to attend CR due to work or personal scheduling issues, or lack the financial means for regular transportation. Personally, patients could misconceive the severity of coronary heart disease and underestimate the effectiveness of CR as a therapy for cardiovascular risk reduction. Some patients try a personal approach to tackle risk factors, but these often lack the multidisciplinary strategies that CR programs utilize. However, CR programs should implement a personalized approach that could motivate patients into committing to long-term behavioral change. On system-level, the lack of referrals, financial issues, and a one-size fits all approach adversely impact CR program uptake. More recently, the COVID-19 pandemic caused major healthcare changes, which affected clinical and post-clinical care. CR centers closed temporarily during peak infection periods and only partially reopened after policy changes. Nevertheless, some patients remained anxious to commute after reopening, causing delays in CR programs. These delays result in poorer uptake, attendance and completion mitigating benefits (12, 13).

A recent systematic review (14) found no differences in cost-effectiveness for the implementation of home-based CR programs. Dalal et al. (15) offered patients free choice or randomization between home-based CR and center-based CR, and found no differences in anxiety and depression, quality of life, or modifiable risk factors. Two systematic reviews suggest that home-based CR was safe, as no important differences in mortality, cardiac events, exercise capacity, modifiable risk factors and quality of life were found (16, 17) when using the ‘Heart Manual’. In a more recent update of the systematic review on home based interventions, these conclusions remain the same (18). Completely or partially delivering component CR programs might tackle some barriers of participation. Some methods of home-based CR delivery commonly start with attending a few exercise sessions on site (19, 20), after which participants are guided through telephone calling (15, 20, 21), are given educational material (22, 23), or are asked to keep logs on their exercise progression (21).

Telehealth, mHealth interventions and home-based CR are class IIb recommendation (24) in the European Society of Cardiology guidelines. Tele monitoring allows for a change in frequency of face-to-face contact, which in turn could replace home visiting. This might benefit the costs of home-based CR by reducing the number of home visits by nurses (22). Moreover, providing remote feedback on goal progression and providing long-term support might be more feasible in cardiac telerehabilitation (CTR), which are essential components of behavioral change interventions and a class I recommendation (25).

Duration of traditional center-based CR programs usually last for a few weeks to a few months. However, adherence to new behavioral change typically starts after four to six months (25), which is a longer period of time than the duration of most center-based CR programs, but well within the timeframe of CTR. CTR could expand on CR core components by integrating tele coaching, social interaction, tele monitoring and e-learnings (3). Previous CTR research mainly focused on improving exercise capacity (26–28). However, focusing on only one of the core components of CR limits the multidisciplinary approach of CR programs, which means a lot of CTR programs exist as an extension of the exercise program of existing center-based CR rather than a complete CR program.

Remote monitoring offers patients regular reminders to engage in exercise or pursue their health goals more consistently. Long-term tele monitoring might sustain behavioral change (29), such as daily physical activity or non-exercise related core components (e.g., nutritional counseling, psychosocial management or tobacco cessation). Moreover, tele monitoring could support behavioral change strategies (e.g., goal setting, self-monitoring, frequent and prolonged contact), which are essential components for any dietary of physical activity behavioral change (25). Consequently, center-based CR often lack some behavioral change strategies that require long-term follow-up or regular positive reinforcement. Currently, it remains unclear who benefits most from CTR programs (29). Whether home-based CR, let alone CTR might benefit uptake of CR among elderly patients was unproven (30). Recent research (31) seems to suggest that mobile guided CR benefits physical activity in the elderly compared to non-participation, which would underline the importance of whether uptake improves in elderly after CTR implementation. Although older adults are less likely to use mobile technology (32), this is a large proportion of the patients in need of CR. The perception of limited digital skills is a challenge for this population (33). In order to implement mobile technology for older adults, new mobile technology could benefit from a comprehensible digital platform that offers the multidisciplinary approach that center-based CR offers. A simple digital platform might positively reinforce patients using visual cues. Fully remote CR programs lack the social components of center-based CR. If this is perceived as such, remote CR might not be appealing for specific subgroups of patients like women (9).

Previous research (7) explored the factors leading to non-participation in a telerehabilitation trial. Results show that factors leading to non-participation in this telerehabilitation trial (7) are associated with a worse cardiovascular risk profile (e.g., high age, a higher prevalence of modifiable risk factors, lower exercise capacity), lower prevalence of high education, lower prevalence of employment, and shorter travelling distance to the nearest hospital. Our hypothesis matches these results i.e., that patients not interested in CTR have a worse cardiovascular risk profile, a lower social economic status, and live closer to the nearest hospital than patients who are interested. The REHAB + study is the first large (>150 patients) multicenter and multinational prospective observational study to evaluate the effect of cardiac telerehabilitation in daily practice. It provides the possibility to gain experience with implementing cardiac telerehabilitation. Our study design differs from previous research by its size, design (observational instead of RCT) and inclusion in different European centers and countries. It provides beneficial information about patient characteristics and results. The aim of the REHAB + trial is to identify which parameters influence the decision to participate in center-based CR, CTR, or decline participation in CR. We will compare quality of life, physical activity, and modifiable risk factors before, during and after program completion. We expect an improvement in quality of life, specifically the physical component, in both center-based CR and CTR.

## 2 Methods

### 2.1 Study design

The REHAB + trial is a multicenter, prospective, matched controlled observational trial with participation from the following centers: Zuyderland Medical Center (Heerlen, The Netherlands), Hospital Virgen de la Victoria de Málaga (Malaga, Spain), and Hospital Universitari Vall d’Hebron (Barcelona, Spain). The medical ethical commission of all participating centers approved the study protocol. Inclusion started in November 2021 and the last patient was enrolled in Februari 2024. The trial is registered at clinicaltrials.gov with registration number NCT05207072.

### 2.2 Study population

Patients eligible for CR who are unwilling to participate in either CTR or center-based CR are allocated to the non-participation category of the study. Patients eligible for the trial are (N)STEMI patients over 18 years old that meet the eligibility criteria to participate in a CR program. Eligibility criteria for CR include the physical ability to safely perform an exercise test. Patients with myocardial ischemia during the exercise test, and judged unsafe for CR participation by a trained cardiologist, are excluded from the trial. Patients are required to have the mental ability to cooperate by following instructions from their coaches and need the proper knowledge to comprehend the native language of the coaches. Patients participating in CR at a different CR center during or after hospitalization are excluded. The research nurse and cardiologist reserve the right to exclude patients deemed unfit for CR due to severe comorbidities. A complete overview of in- and exclusion criteria is found in Table 1.

**Table 1:** In- and exclusion criteria for cardiac (tele) rehabilitation.

Inclusion criteria	
CR	Recent myocardial infarction [(N)STEMI only]
CR	Signed written informed consent
CTR only	Smartphone capable of installing REHAB + application
Exclusion criteria	
CR	Contra-indication for CR (such as risk for safety and limited lifespan)
CR	Mental impairment leading to an inability to cooperate
CR	Severe impaired ability to exercise (including an inability to safely perform the exercise test)
CR	Insufficient knowledge of the native language
CR	Current participation in CR (at CR center or elsewhere)
CR	Participation in a CR program elsewhere after index myocardial infarction

### 2.3 Treatment allocation

**Figure 1:**
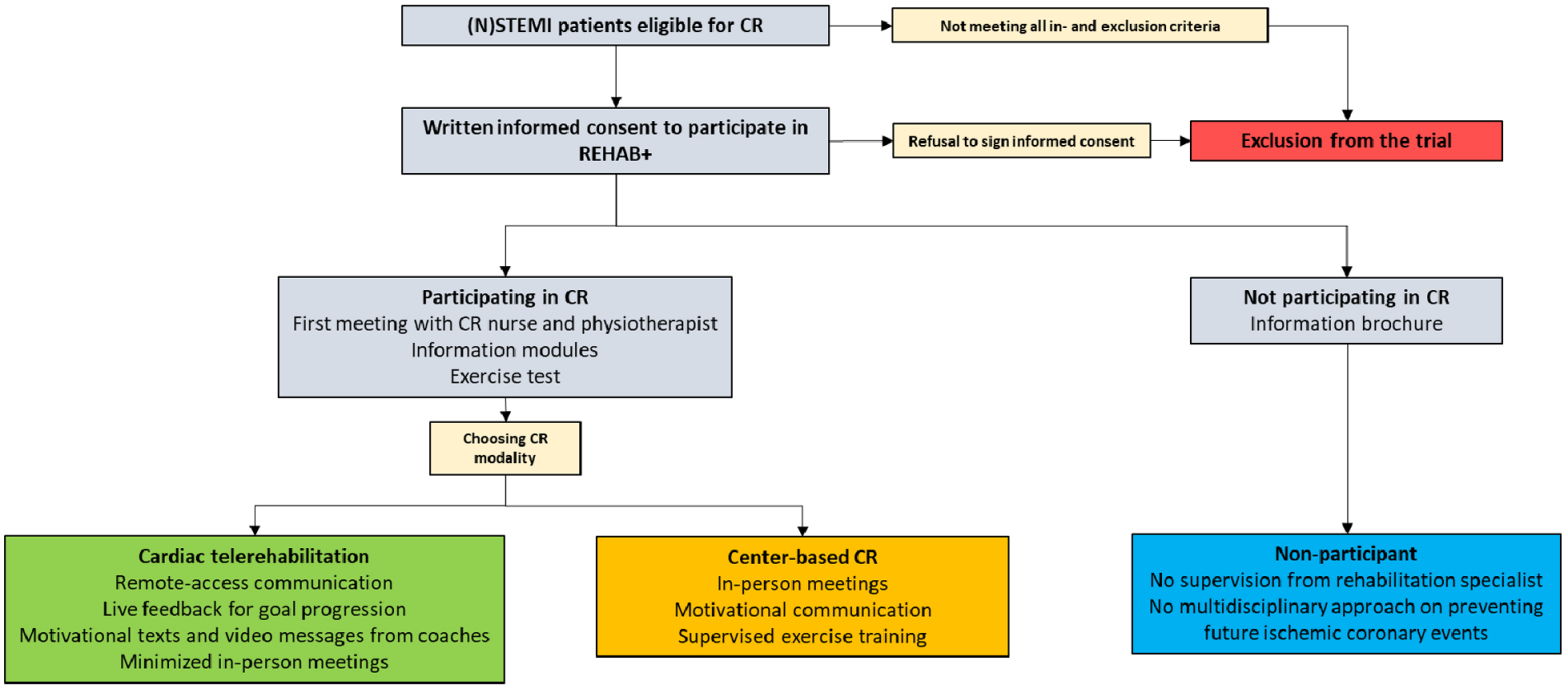
Flowchart of the inclusion procedure.

The coaches and investigators are explicitly instructed to refrain from advising patients on their choice between CTR and center based CR to avoid bias. However, coaches and investigators are allowed to advise patients to participate one of either CR programs, as that is common practice. Providing neutral information about the advantages and disadvantages of both treatment options is allowed. After signing, all participants are free to choose between CTR and center-based CR. Non-participants are registered as such, provided they meet eligibility criteria and sign informed consent. The patients meet with the nurse after an exercise test to inform them of their allocation choice. The flowchart of the inclusion procedure is found in Figure 1.

### 2.4 Cardiac rehabilitation

Cardiac rehabilitation nurses screen potential participants during hospitalization. Preferably, they make first contact during the hospitalization phase to motivate patients to participate in CR and inform them about the content of the program. Afterwards, the nurses contact patients by phone after discharge to arrange a first appointment. The aim is to start CR as soon as possible, usually within 3–4 weeks after discharge from the hospital after PCI and 6 weeks after CABG. All patients will start the CR program with an exercise test to ensure safe rehabilitation and to provide information on the exercise capability of the patient. The nurse provides patients with health modules from the library of content to tackle modifiable risk factors (e.g., nutrition, exercise, psychology, and the societal impact of cardiovascular disease). A specialized psychologist works with patients that experience anxiety after their myocardial infarction. A social worker discusses any problems experienced at home.

### 2.5 Center-based cardiac rehabilitation

The exercise-training program of the center-based CR program lasts for approximately 6–8 weeks depending on the needs of the patients. The educational, psychological, and lifestyle parts of the CR program are usually completed within these 6–8 weeks. The completion of center-based CR is marked by the completion of the exercise-training program and by a wrap-up meeting with the nurse. The nurse will ask to evaluate the program, reflect on the newly learned health behavior and motivates patients to continue with healthy behavior change at home.

### 2.6 Cardiac telerehabilitation

#### 2.6.1 REHAB + application

**Figure 2:**
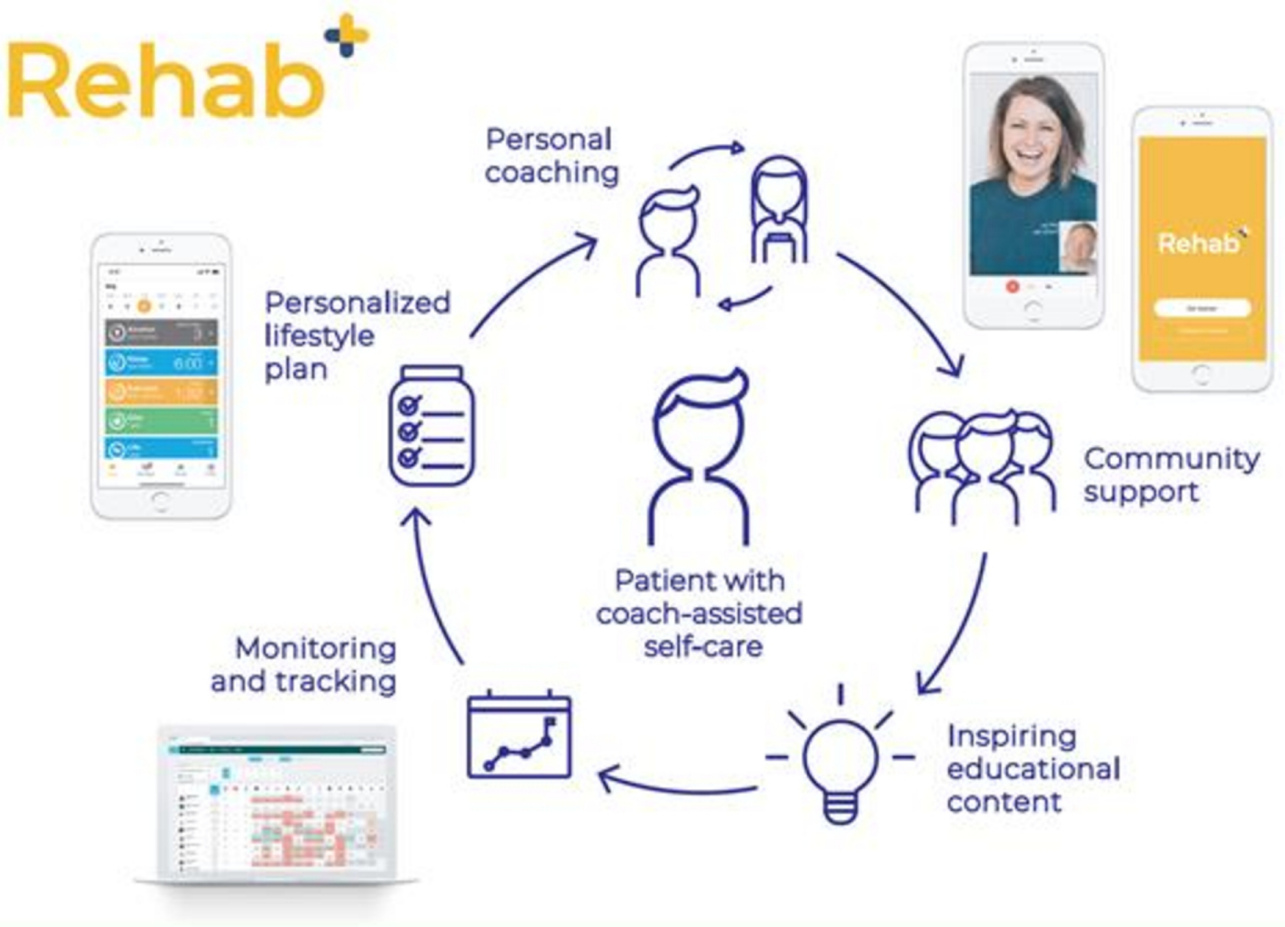
Overview of cardiac telerehabilitation via the liva app.

The CTR program start with the installation of the REHAB + app and instructions on how to use the app (Livahealthcare.com). The REHAB + app provides participants and coaches with asynchronous, remote-access communication via text or video messaging (Figure 2). Coaches can share additional health information from the comprehensive library of content to aid health goal progression. Coaches can motivate or support participants depending on their goal progression. Participants can upload and monitor health status information [e.g., mode of physical activity, rate of perceive exertion (BORG-score), blood pressure, glucose levels, eating habits, sleeping habits and smoking habits]. The REHAB + app receives physical activity input from a wearable heart rate device and/or pedometer. Each participating center is free to make use of clinically approved wearables they see fit for supporting remote exercise training supervision. Participants are allowed to make use of a wearable heart rate device of their own if it matches with the requirements of the app. The time and effort the coaches provide each patient is mainly concentrated during the first few weeks of CR. Moreover, when patients show sufficient goal progression and self-efficacy, time and effort provided by the coaches will be toned-down. The CTR program will provide tele monitoring for 48 weeks, after which the CTR program ends and patients are motivated to continue their newly learned health behavior and goal progression at home.

#### 2.6.2 Exercise

The physiotherapist creates a personalized exercise program that fits the exercise capability of the participant. We minimized the number of in-person training sessions (max 2), only explaining the essentials and guiding participants in the use of the REHAB + app. Each participating center is free to structure their own exercise-training program in adherence to European and national guidelines. Participants are encouraged to perform the exercises taught by their physiotherapist at home. Patients receive advice on other modes of exercise at home from the physiotherapist (e.g., walking or cycling, and the duration and intensity of the exercise). The REHAB + app allows participants and coaches to keep track of their perceived intensity during exercise (via BORG-scale). Participants can also keep track of the frequency in which they exercise in the 48 weeks they can use the REHAB + app.

### 2.7 Data collection

Data are collected at program initiation (T0), three months after initiation (T1) and twelve months after initiation (T2). Data collection from non-participants only include baseline characteristics at T0. Researchers collect data from the electronic health record. Exercise-related data will be collected by Liva (Livahealthcare.com). Data will be made available after all participants completed their CTR program. Participants receive questionnaires by e-email or by postal mail. Participant data is pseudo anonymized. The SPIRIT diagram is found in Table 2.

**Table 2:** SPIRIT diagram for REHAB+ study.

	Study period			
	Enrolment	Allocation	Post-allocation	Close-out
Timepoint^a^	*-t_1_*	0	*t_1_*	*t_2_*
Enrolment:				
Eligibility screen	(X)			
Informed consent	(X)	(X)		
Allocation		(X)		
Interventions:				
Center-based cardiac rehabilitation		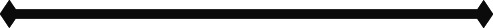		
Cardiac telerehabilitation		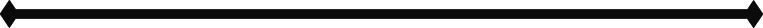		
Non-participation	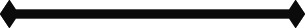			
Assessments:				
Baseline characteristics^b^		(X)		
Medication, blood pressure, BMI		(X)	X	X
Blood sampling^c^		X	X	X
Exercise test		X		X
Adherence and compliance			X	X
Adverse (cardiac) events			X	X
Fägerstrom test		X	X	X
SF-36 questionnaire		X	X	X
IPAQ-SF		X	X	X
eHIQ^d^			X	

Cells marked with (X) are data gathered for center-based CR, CTR and non-participants of CR. Cells marked with X are data gathered for both center-based CR and CTR. SF-36 36-Item Short Form Health Survey, IPAQ-SF International Physical Activity Questionnaire-Short Form, eHIQ e-Health Impact Questionnaire.

^a^ -t1= Period between hospitalization and start cardiac rehabilitation, t0 = start cardiac rehabilitation, t1 = three months, t2 = twelve months.

^b^ Demographics, medical history, risk factors, coronary angiography outcome, mode of revascularization, type of acute coronary syndrome, left ventricular ejection fraction and twelve lead electrocardiogram.

^c^ Lipid profile, kidney function and Hb1Ac.

^d^ Participants in the cardiac telerehabilitation group receive an extended questionnaire containing specific questions about the REHAB + app.

The main source of baseline data extraction is the electronic medical file, besides questionnaires. Baseline characteristics include age, sex, ethnicity, employment status, marital status, living situation and distance from the hospital. Traditional risk factors data collection include a medical history of hypertension, hypercholesterolemia and diabetes (including HbA1c), current smoking status and family history of cardiovascular disease. Moreover, a researcher from the research team extracts the medical reason for CR participation, medical history relevant to cardiovascular diseases and the current use of cardiovascular medication. Finally, information from focus tests, such as heart ultrasound (LV function), coronary angiography (with or without modes of revascularization) and electrocardiography will be interpreted and obtained by a medical doctor from the research team. Finally, results from the exercise test include blood pressure, peak blood pressure, METS score and ECG changes at peak exercise capacity.

The REHAB + app monitors adherence and compliance of CTR participants. Adherence is defined as the number of dropouts and the reason for drop-out in relation to participants completing CTR. Compliance is defined as the number of logins and the proportion of planned exercise sessions completed, as well as which sessions are completed (i.e., exercise, quitting smoking). Information from i.e., the heart rate monitor, goal progression, data input by the participant (e.g., BORG scale) and interaction with the coaches will be logged in the app.

#### 2.7.1 Questionnaires

Participants evaluate quality of life at baseline, three months and twelve months using the 36-Item Short Form Health Survey (SF-36) version 2.0. Each participating center used a validated version translated to the local language (34, 35). The questionnaire provides a score on eight domains. These translate to a physical component score and a mental component score. Domains mostly related to the physical component score has the most pure interpretation for chronic medical conditions (36).

A coach and cardiologist assess physical fitness at baseline using an exercise test. The participant completes the exercise test by exerting their maximal workload. However, the test stops prematurely if the participant exhibits angina. Physical activity is evaluated for all participants using the International Physical Activity Questionnaire-Short Form (IPAQ-SF) (37, 38). The IPAQ-SF is a 7-item questionnaire, which allows the calculation of metabolic equivalents (METs score) using self-reported physical activity intensity and frequency.

The modifiable risk factors are documented during hospitalization, from which baseline data on the use of tobacco products is extracted. Patients fill out the Fagerström test, testing nicotine dependency at baseline, three months and twelve months. The Fagerström test is a validated (39, 40), 5-item questionnaire with a scoring system that categorizes dependency on nicotine in four categories (barely dependent, to lightly dependent, to moderately dependent, to strongly dependent).

CTR participants receive the e-Health Impact Questionnaire (eHIQ) at baseline, three months and twelve months, evaluating their previous general health experience (11-item questionnaire) and the digital health experience of the REHAB + app (15-item questionnaire). Center-based CR participants only receive the general health experience section. The eHIQ is validated for Dutch participants (41). An official translation agency translated the eHIQ into Spanish for the Spanish centers with permission of the original authors.

### 2.8 Sample size calculation

The sample size calculation is based on an expected increase in quality of life after one year, specifically the physical component score change of the SF-36 questionnaire V2.0. Previous research (42) found baseline quality of life physical component score of 48.4 + - 5.8 at program initiation and 53.3 + - 3.9 after 12 months. We estimated an increased score to 55 + - 7.0 for CTR patients. The sample size calculation, using the Hotelling Lawley Trace test, resulted in a sample size of n = 268 per group (power = 0.8 and alpha = 0.05). Assuming 10% withdrawal or incomplete data, we aim for n = 300 participants per group. We expect a 1:2 ratio of participants choosing CTR vs. center-based CR. We aim to continue inclusion until 300 participants are included in the CTR group, with enough participants to match in center-based CR (600). This will likely result in 300 participants per participating CR center (100 participants choosing CTR and 200 participants choosing center-based CR).

### 2.9 Statistical analysis

Continuous data will be analysed with the independent T-Test (or Mann-Whitney U test for non-normal distribution of data). Categorical data will be analysed using the chi-squared test (two groups) or ANOVA (> two groups). The One-way ANOVA test (or Kruskal Wallis test for non-normal distribution of data) will be used to analyse >two continuous data categories. The impact of multiple variables on allocation is analyzed using the multinomial regression analysis. All statistical tests are considered significant at P < 0.05. Statistical analysis will be performed on the latest version of IBM SPSS Statistics or R.

## 3 Discussion

The REHAB + trial is a large (> 150 CTR patients), multicentre and international study and is unique in the fact that it allows participants to choose between center-based cardiac rehabilitation and CTR. This design closely resembles clinical practice, providing valuable insights into patient decision-making. Recently, the American Heart Association recommended that future research on behavior change strategies should more closely focus on routine clinical practice, as opposed to efficacy trials (25), aligning with the approach of the REHAB + trial. Telerehabilitation has the potential to increase uptake of CR by overcoming barriers such as travel time and aligning with specific patient preferences. The provision of longer follow-up times offers patients the opportunity sustained engagement, which could tackle the typical decline of behavioural change that could take 4–6 months to internalize (25). Consequently, the implementation of a tele health strategy in CR requires protocol retailoring of existing center-based CR. For one, tele health allows for longer follow-up of behavioural change strategies and exercise monitoring. Patients are free to select convenient moments to exercise or work on their behavioural change strategies. Thus, CTR programs require a different approach on the timing, duration, frequency and intensity of the program. Typically, center-based CR lasts 6–8 weeks, which is different from our CTR program. This program involves fewer on-site visits, replacing these visits with remote monitoring, mostly concentrated in the first few weeks of CR. Additionally, participants can use the application for up to 48 weeks for habit formation and for monitoring goal progression. Since the follow-up period in our telerehabilitation program is longer than the center-based CR program, there might be less risk of relapse to old habits, which could influence outcomes on quality of life, favouring CTR. Since CTR is offered as an alternative in routine clinical practice, not as a replacement for center-based CR, we foresee no negative consequences for our patients if results favour CTR.

A comprehensive systematic review on the effectiveness of telerehabilitation focussing on exercise therapy (43) concluded that telerehabilitation in multiple fields could be comparable or better than conventional methods of rehabilitation, even suggesting the possibility of better mortality outcomes in cardiac rehabilitation. Huang et al. (2015) drew a more conservative conclusion on outcomes such as mortality, adverse events, modifiable risk factors, exercise capacity, health related quality of life and psychological state, suggesting that telehealth interventions in CR are non-inferior to center-based CR in low to moderate risk CAD patients (26). A more recent systematic review on safety outcomes reported low incidence of reported SAEs and re-hospitalization in remotely delivered CR in interventions lasting at least 12 weeks (44) suggesting it as a safe alternative to center-based CR. Another systematic review (45) suggests CTR being cost-effective compared to traditional center-based CR programs. However, the studies included in the review are heterogeneous and relatively small in sample size. Lastly, Antoniou et al. (2022) suggests that the use of wearable sensors in home-based positively influence cardiac risk factors (46). Additionally, objective monitoring is more accurate in depicting actual physical activity, challenging subjective determination of physical status.

The SmartCare-CAD trial designed a novel cardiac telerehabilitation intervention, using cognitive-behavioural strategies in the implementation of cardiac telerehabilitation as relapse prevention (47). Patients were randomized between the novel CTR intervention and the existing center-based CR program. In a prospective sub-analysis (7), the SmartCare-CAD trial evaluated demographic predictors for trial participation. Out of 699 patients, 399 were uninterested in trial participation. Most patients lacked interest in digital health (26%) or preferred center-based CR to CTR (21%). REHAB + resembles SmartCare-CAD by analyzing factors that evaluate demographic reasons for non-participation, in addition to CTR or center-based CR participation. However, REHAB + is an observational trial, which could more closely resemble daily clinical practice.

The Telerehab III trial (48) assessed the effectiveness of additional telerehabilitation after center-based CR. All patients (70 controls, 70 intervention) received center-based CR. The intervention group received an additional six-month tele-monitoring program halfway through the center-based CR program. The authors observed initial improvements of VO2 peak and daily physical activity six weeks into the program for both groups, which decreased in the control group after 24 weeks. Since adherence to newly learned health behaviour typically declines after 4–6 months (25), the value of additional cardiac telerehabilitation effectively supports patients in long-term health behavioural change. In an additional analysis, the addition of CTR after center-based CR was evaluated cost-effective, while improving exercise capacity, health behavioural change and quality of life (49). In the timing of intensive tele monitoring, the REHAB + trial is different from Telerehab III. High intensity support is provided at the start of the program, which slowly decreases over time according to individual needs.

The REMOTE-CR trial (50) randomized patients for exercise based cardiac telerehabilitation or a center-based CR exercise program. Both groups received 12 weeks of strenuous coaching with three exercise moments with their coach per week. REMOTE-CR confirmed their primary aim, which was to achieve comparable levels of VO2max in both groups after 12 weeks of supervised exercise. The implication is that remote exercise programs could be implemented to achieve comparable exercise-related outcomes in patients.

A study by Khadanga et al. (2021) (51) assessed predictors for participation in a center-based CR program as well. A total of 378 eligible patients were approached to sign consent to use their data. Of these patients, 294 enrolled in the study and 175 participated in at least on session of CR. The authors found that the main barriers for non-participation were a lack of interest in CR (23%), transportations issues (22%) and no referral or recommendation by their clinician (18%). The REHAB + trial will offer patients the additional choice of CTR, which may tackle some of these barriers. CTR limits the number of times patients have to visit the hospital. In turn, transportation issues might play a less significant role, as patients could ask their social circle for transportation or take public transportation for the few times they need to visit the CR center. Patients who lack the interest to participate in center-based CR due to group lessons or patients that feel like they can do the exercises at home could prefer CTR. CTR might therefore be a valid option to increase participation for CR in general.

### 3.1 Limitations

Although the implementation of CTR might be as (cost-) effective as center-based CR, there could be a learning curve at the start of this newly implemented CR program. Center-based CR and CTR could vary in frequency, intensity, duration and initiation of the program. However, this could be a strength when patients are allowed to compare the programs and choose which one fits them best. It could lead to improved participation in CR.

The coaches of the REHAB + application will use the remote communication feature to frame motivational texts or video’s to benefit long-term uptake of the exercise program. The ability to track behavioral health goals in CTR is a feature that lacks in center-based CR. The frequency of in-person exercise training sessions might initially favor center-based CR short-term on quality of life and exercise goals. However, the ability to track health behavioral goals and the ability to use the application for over nine months might favor CTR long-term.

Local differences may occur due to the unique features that each CR center might implement additionally to local or international guidelines. A learning curve for CR centers without experience with CTR is expected to occur. The nature of treatment allocation of REHAB + results in selection bias, which does resemble daily clinical practice. There is also the possibility that the expected ratio of 1:2 center-based CR and CTR will not be met, as participants have the freedom to choose their CR modality.

Due to the nature of the study, in which clinical practice is closely evaluated, the use of a follow-up exercise test to evaluate physical activity after CR was not feasible. Therefore, the choice for a less rigorous method of physical activity evaluation was chosen, namely by questionnaire which could have recall bias. Participants will have a baseline exercise test to evaluate their physical condition.

## 4 Conclusion

The REHAB + study will provide new insights on patient preference for CR programs and patient characteristics associated with those preferences. REHAB + will compare the effectiveness based on quality of life, physical activity and positive changes in modifiable risk factors between center-based CR and CTR.

## Data Availability

The raw data supporting the conclusions of this article will be made available by the authors, without undue reservation.
